# Reply to Gringras et al. Comment on “Paditz et al. The Pharmacokinetics, Dosage, Preparation Forms, and Efficacy of Orally Administered Melatonin for Non-Organic Sleep Disorders in Autism Spectrum Disorder During Childhood and Adolescence: A Systematic Review. *Children* 2025, *12*, 648”

**DOI:** 10.3390/children12111564

**Published:** 2025-11-18

**Authors:** Osman S. Ipsiroglu, Hans-Jürgen Gober

**Affiliations:** 1 Interdisciplinary Sleep Program, Sleep/Wake-Behaviour Clinic, Departments of Pediatrics & Psychiatry, University of British Columbia, Vancouver, BC V6T 1Z4, Canada; 2 Department of Pediatrics, Medical University of Vienna, 1090 Vienna, Austria; 3 Institute for Sleep and Wake Research, 1140 Vienna, Austria; 4 Department of Pharmacy, Kepler University Hospital, Neuromed Campus, Johannes Kepler University, 4020 Linz, Austria; hans.gober@kepleruniklinikum.at

We read, with great interest, the Letter to the Editor of Drs. Gringras’, Malow’s and Schröder’s comments [1] on the manuscript by Paditz et al. *The Pharmacokinetics, Dosage, Preparation Forms, and Efficacy of Orally Administered Melatonin for Non-Organic Sleep Disorders in Autism Spectrum Disorder During Childhood and Adolescence: A Systematic Review* [2] and the response by Paditz et al. [3].

Paditz et al. (2025) conducted a formal review of randomized clinical trials (RCTs) for melatonin in children and adolescents with autism spectrum disorder (ASD) [2] with the aim to identify unmet needs in generating evidence for long and short acting formulations of melatonin in this patient group. The rationale for inclusion and exclusion of certain RCTs in Paditz’s review was subject to Gringras’ critique [1] and further explained by Paditz et al. in their response [3].

The discourse boils down to the question of whether the therapeutic action of melatonin is affected by neurodevelopmental, behavioural, and psychiatric comorbidities frequently associated with ASD and whether rapid- or controlled-release formulations are preferable. While the complexity of this question has not been entirely clarified by previous studies, for a clinician this debate unveils another unresolved question: which melatonin-based products have been approved by the European Medicines Agency (EMA) in the European Union (EU), and what data supported their authorization. On this point, Paditz et al. and Gringas et al. have contradictory perceptions [1,2,3].

Whereas melatonin is sold over the counter as a dietary supplement in North America, information on market authorization is important for prescribers in Europe and the UK where melatonin is handled as a pharmacological agent. To better inform clinical prescribing practice, we reviewed the data available on the EMA website and found only three melatonin products; all of them are controlled-release/retard formulations marketed by Neurim Pharmaceuticals Ltd. These products received EMA market authorization between 2007 and 2022 (Table 1). Rapid-release melatonin formulations have not been approved by the EMA, but several products have been approved by national regulatory authorities in individual European countries.

The historical approval data shows that Circadin^®^ (approval 2007) and Melatonin Neurim^®^ (generic formulation of Circadin^®^, approval 2022) have been authorized for treatment of primary insomnia based on studies in adults [4,10]. Pediatric studies with controlled-release melatonin by Wasdell et al. [11] and Gringras et al. [6] were pivotal for supporting the subsequent approval of Slenyto^®^ for use in children with ASD (approval 2018). Further, Slenyto^®^ market authorization was subsequently extended to include use in other neurodevelopmental diagnoses, such as neurogenetic disorders (2024) and primary attention deficit hyperactivity disorder (ADHD) (2025). Interestingly, the authorization for ADHD refers to results of the original study by Gringras et al. [6] and a derived sub-group analysis of pharmacokinetics in 16 patients with ASD but not with primary ADHD (*Slenyto^®^ Product Information. Pharmacokinetic properties: Absorption In the paediatric population comprising 16 ASD children ages 7–15 years old suffering from insomnia, following Slenyto 2 mg (2 × 1 mg mini-tablets) administration after a standardized breakfast, melatonin concentrations peaked within 2 h after administration and remained elevated for 6 h thereafter with a Cmax (SD) of 410 pg/mL (210) in the saliva*) [9]. Based on this information, we can conclude that there is no available separate pharmacokinetics data for the ADHD subgroup, which is an important limitation, considering that endogenous melatonin kinetics might be different in ADHD [12].

Creating evidence is a challenging task. Evidence generated by numerous studies for the efficacy of rapid release melatonin in primary ADHD could not be utilized to obtain EMA authorisation. A recent meta-analysis might give the answer of the why: Edemann-Callesen et al. found eight RCTs testing melatonin in children and adolescents (6–19 years of age) for primary insomnia, encompassing 419 participants in total [13]. Five of the eight RCTs were conducted with rapid release melatonin; however, the formulation of the remaining three RCTs is unknown (!). This uncertainty supports our concerns regarding study-design-related challenges, which also does not make the task of the EMA easier. These unresolved questions regarding controlled- and fast-release melatonin products and the multidimensional complexity of insomnia in ASD and ADHD with all their neurodevelopmental (co-)morbidities makes creating evidence via formal clinical trials a Sisyphean task.

Personalized medicine using N-of-1 protocols with standardized trial designs, and thus allowing meta-analysis, is an innovative approach for evidence generation which might be considered by scientists and pharmaceutical companies to overcome trials and tribulations in the present melatonin conundrum [14,15]. The necessary creation of standardized sets of outcome measures would also allow the utilization of new insights into phenotypic presentations such as sleep hyperhidrosis and restless legs syndrome [16,17,18].

Meanwhile, for the practising clinician, the *Melatonin Statement* of the International Pediatric Sleep Association [19] and Melatonin Working Group of the German Sleep Society [20,21] should serve as the guideline, indicating that, *children and adolescents with non-organic insomnia melatonin should be dosed according to age- and the underlying primary diagnosis after a careful clinical assessment*, and remain the generic common denominator for decision making in day-to-day clinical practice. Changing perspectives and investigating differential diagnostic considerations before prescribing any drugs (including melatonin) is consistent with both bodies’ recommendations and the goals of personalized medicine [19,20,21].

## Figures and Tables

**Table 1:** Provides an overview of the available EMA-approved retard formulations (by Neurim Pharmaceuticals Ltd., Tel Aviv, Israel), their approval dates, designation for patient populations, and pharmacokinetics data. Note that only one formulation, Slenyto^®^, has been approved for application to children and adolescents and there are no rapid-release melatonin formulations approved by the EMA.

Product	Formulation	Year of Market Authorization	Approved Age	Therapeutic Indication	Pharmacokinetics
**Circadin^®^**	Retard release	2007	55 y	Short-term treatment of primary insomnia in adults. Ref. [4]	Tmax: 3 h T½ (ret.): about 3 h after reaching Tmax Ref. [5]
**Slenyto^®^**	Retard release, small tablets thus more convenient for children	2018	2–18 y (ASD) 6–17 y (ADHD)	Insomnia in ASD (2018) Neurogenetic disorders with insomnia (2024) Refs. [6,7,8] Insomnia in ADHD (2025) Ref. [9]	Tmax: 2 h T½ (ret.): 3.5–4 h Melatonin remains elevated for 6 h after reaching Tmax Ref. [9]
**Melatonin** **Neurim^®^** **(generic formulation of Circadin^®^)**	Retard release	2022	≥55 y	Short-term treatment of primary insomnia in adults Refs. [4,10]	Tmax: 3 h T½ (ret.): about 3 h after reaching Tmax Ref. [5]

## Abbreviations

The following abbreviations are used in this manuscript:

RCT	randomized clinical trial
ASD	autism spectrum disorder
EMA	European Medicines Agency
EU	European Union
ADHD	attention deficit hyperactivity disorder
